# Mortality associated with alternative primary healthcare policies: a nationwide microsimulation modelling study in Brazil

**DOI:** 10.1186/s12916-019-1316-7

**Published:** 2019-04-26

**Authors:** Davide Rasella, Thomas Hone, Luis Eugenio de Souza, Renato Tasca, Sanjay Basu, Christopher Millett

**Affiliations:** 1 Instituto de Saúde Coletiva, Universidade Federal da Bahia, Salvador, Bahia Brazil; 2 Public Health Policy Evaluation Unit, Department of Primary Care and Public Health, School of Public Health, Imperial College London, London, UK; 3 Pan-American Health Organization/World Health Organization Country Office for Brazil, Brasilia, Brazil; 4 Center for Population Health Sciences, School of Medicine, Stanford University, Stanford, California USA; 5 Center for Primary Care and Outcomes Research, School of Medicine, Stanford University, Stanford, California USA; 6 Center for Epidemiological Studies in Health and Nutrition, University of São Paulo, São Paulo, Brazil; 7 Center for Primary Care, Harvard Medical School, Boston, Massachusetts USA

**Keywords:** Primary care, Health system financing, Brazil, Mortality, Microsimulation

## Abstract

**Background:**

Brazil’s *Estratégia Saúde da Família* (ESF) is one of the largest and most robustly evaluated primary healthcare programmes of the world, but it could be affected by fiscal austerity measures and by the possible end of the *Mais Médicos* programme (MMP)—a major intervention to increase primary care doctors in underserved areas. We forecast the impact of alternative scenarios of ESF coverage changes on under-70 mortality from ambulatory care-sensitive conditions (ACSCs) until 2030, the date for achievement of the Sustainable Development Goals (SDGs).

**Method:**

A synthetic cohort of 5507 Brazilian municipalities was created for the period 2017–2030. A municipal-level microsimulation model was developed and validated using longitudinal data and estimates from a previous retrospective study evaluating the effects of municipal ESF coverage on mortality rates. Reductions in ESF coverage, and its effects on ACSC mortality, were forecast based on two probable austerity scenarios, compared with the maintenance of the current coverage or the expansion to 100%. Fixed effects longitudinal regression models were employed to account for secular trends, demographic and socioeconomic changes, healthcare-related variables, and programme duration effects.

**Results:**

Under austerity scenarios of decreasing ESF coverage with and without the MMP termination, mean ACSC mortality rates would be 8.60% (95% CI 7.03–10.21%; 48,546 excess premature/under-70 deaths along 2017–2030) and 5.80% (95% CI 4.23–7.35%; 27,685 excess premature deaths) higher respectively in 2030 compared to maintaining the current ESF coverage.

Comparing decreasing ESF coverage and MMP termination with achieving 100% ESF coverage (Universal Health Coverage scenario) in 2030, mortality rates would be 11.12% higher (95% CI 9.47–12.76%; 83,937 premature deaths). Reductions in ESF coverage would have stronger effects on mortality from infectious diseases and nutritional deficiencies and would disproportionately impact poorer municipalities, with the concentration index for ACSC mortality 11.77% higher (95% CI 0.31–22.32%) and also ending historical declines in racial health inequalities between white and black/pardo Brazilians.

**Conclusions:**

Reductions in primary healthcare coverage due to austerity measures are likely to be responsible for many avoidable deaths and may preclude achievement of SDGs for health and inequality in Brazil and in other low- and middle-income countries.

**Electronic supplementary material:**

The online version of this article (10.1186/s12916-019-1316-7) contains supplementary material, which is available to authorized users.

## Introduction

Universal Health Coverage (UHC) is high on the global health agenda and a key target included among the Sustainable Development Goals (SDGs) [1, 2]. The 40th anniversary of the Alma-Ata declaration [3] this year is a timely reminder that Primary Health Care (PHC) is considered essential to achieve UHC [4]. Evidence suggests that health systems with well-developed PHC are best placed to deliver an affordable package of care with greater population coverage, producing better and more equitable health outcomes [5–7].

Progress towards UHC is threatened by constrained public financing resulting from economic crises in some middle-income countries. An important example is Brazil, where an economic crisis has endured since 2014. The federal government has introduced a long-term programme of fiscal austerity limiting public expenditure for social welfare programmes (Box 1). These austerity measures are reducing federal spending on the *Sistema Único de Saúde* (SUS), Brazil’s public healthcare system [8–10].

The *Estratégia Saúde da Família* (ESF; Family Health Strategy) is the primary vehicle for achieving UHC within the SUS and is one of the largest PHC programmes of the world [11]. ESF coverage has expanded from 6.6% in 1998 to 63.7% in 2016 (covering 123 million people). Since 2013, the ESF has been strengthened through the *Mais Médicos* (More Doctors) programme (MMP) aiming to address under-provision of doctors in remote and deprived urban areas. Approximately 18,000 doctors were installed in ESF clinics in underserved locations, with the majority coming from Cuba [12] (Box 2). The ESF encompasses key principles of PHC including community-based care, multi-disciplinary teams, and a focus on prevention and health promotion [13]. Prior studies associated expanding ESF coverage with reduced infant mortality [14], adult mortality from conditions amenable to health care [15, 16], and health inequalities [17]. Further expansion of the PHC coverage within Brazil would be expected to improve efficiency and equity of the health system [18].

Here, we aim to rigorously forecast potential health impacts of alternative ESF coverage scenarios until 2030. Four future scenarios, representing a range of policy options in Brazil, are modelled:

1. A status quo scenario, where municipal ESF coverage (mean municipal coverage 80.4% in 2016) remains the same. This scenario assumes no deterioration in ESF effectiveness (on health outcomes), for example, from reducing services provided or quality whilst maintaining coverage figures.
2. A contracting ESF coverage scenario related to the current austerity measures, where municipal ESF coverage declines proportional to the federal reductions in per capita health expenditure as per the constitutional amendment [19, 20]. These declines in ESF coverage are based on published forecasts [8], whilst a range of possible declines is tested in sensitivity analyses (Additional file 1).
3. A contracting ESF coverage as above and termination of the MMP, which could end in 2019 due to the lack of political support from the new government [21]. This scenario additionally models specific ESF coverage declines in municipalities where *Mais Médicos* doctors operate.
4. A UHC scenario, where ESF coverage increases to 100% in all municipalities by 2030, reflecting SDG 3, target 3.8 [2]. Expansion of the ESF represents the most effective means to achieving UHC in Brazil [11].

Mortality from ambulatory care-sensitive conditions (ACSCs) was evaluated as health outcomes given evidence of reductions following ESF expansion [17]. ACSCs are conditions which should be preventable with access to quality and timely primary care, and are based on a list of ICD-10 codes published by the Brazilian Ministry of Health [22] (text 3, Additional file 1). They include infectious diseases, nutritional deficiencies, asthma and chronic-obstructive pulmonary disease (COPD), cardiovascular disease, diabetes, epilepsy, and gastric ulcers for those under 70 years of age, also defined as premature deaths [17, 22]. Despite that the list of ACSC was originally created for hospitalizations, it has been used in mortality studies [16, 17] because it is preferred to amenable mortality lists not specific to the Brazilian context. Additionally, potential future inequalities are modelled both *between* municipalities in terms of ACSC mortality rates and *within* municipalities in terms of racial health inequalities between black/*pardo* and white ACSC mortality rates, given Brazil’s high and persistent socioeconomic and health inequalities and the tenth SDG, which aims to reduce inequalities within and among countries [2].

Box 1: Federal austerity and new funding mechanisms for primary health care in Brazil In 2016, the Brazilian government approved a constitutional amendment limiting the growth of federal expenditures. Constitutional Amendment 95 (EC95), abolished minimum federal expenditures on health, and, until 2037, limits growth in federal expenditures on social security, education, social assistance, and health care to inflation. This limits any real growth in federal expenditure on public health care and ignores population growth or increases in health needs [19, 23, 24]. Additionally, as GDP grows and tax revenues rise, there can be no further increases in federal health expenditures over inflation. In 2016, federal expenditure on health was 1.71% of GDP, but under these budgetary limits with annual inflation of 1%, 2%, or 3%, it is predicted to fall respectively to 1.22%, 1.01%, and 0.84% of GDP in 2036 [8]. Given a predicted 10% increase in the Brazilian population by 2036, per capita federal expenditure on health is estimated to fall from R$ 446 in 2017 to R$ 411 in 2036 (in 2016 R$s) [8] (text 2, Additional file 1). In Brazil, 5570 municipal governments are responsible for the provision of public PHC but rely heavily on transfers for primary care from the federal budget. Furthermore, new funding mechanisms introduced in 2018 abolish earmarked funds for ESF increasing municipal health mangers’ discretion in funding services [25–28].

Box 2: The *Mais Médicos* programme In 2013, Brazil initiated the *Programa Mais Médicos* (MMP) (More Doctors programme) to expand the number of doctors in underserved areas. Three components of the programme were initiated: (i) a large-scale recruitment of foreign doctors—mainly from Cuba—through an agreement between Brazil and Cuba coordinated by the Pan American Health Organization (PAHO); (ii) a re-orientation of medical education towards primary care and the opening of new medical schools; and (iii) funds for refurbishment and renovations of ESF facilities. The MMP has facilitated a large increase in ESF doctors—approximately 18,000 doctors are currently participating—with ESF coverage in participating municipalities increasing from 77.9% in 2012 to 86.3% in 2015 [12]. Preliminary findings suggest the programme has reduced avoidable hospitalizations, increased user satisfaction, and improved service quality [12, 29–31].

## Methods

We created a synthetic cohort of 5507 municipalities for the years 2017–2030 using municipal-level discrete-time microsimulation modelling [32] with a longitudinal dataset of municipalities already used in retrospective PHC impact evaluations [16, 17]. Microsimulation forecasting allows the modelling of individual-specific characteristics, using individual-specific previous trends, associated outcome probabilities, subpopulation effects, correlation structures between variables, and non-linear effects.

The microsimulation was based on the conceptual model, the cohort structure and the parameters of a previous retrospective study on the impact of ESF on mortality from ACSC [17]. As in this retrospective analysis, longitudinal fixed effects regression methods were used [34], with as independent variables the same municipal-level demographic and socioeconomic factors, including the effects of healthcare system variables and of large-scale public interventions—such the Brazilian conditional cash transfer Bolsa Familia Programme (BFP)—to predict changes in future ACSC mortality. The forecast scenarios of economic crisis and fiscal austerity were derived from a validated model employed to study the effects of socioeconomic changes and policy options in Brazil and used in a recently published study to simulate under-5 mortality scenarios [33]. The modelling approach was undertaken in two stages: (i) the generation of a synthetic cohort of municipalities with independent municipal-level variable values up to 2030, for BFP and ESF coverage from real data up to 2016 and forecast projections according to scenarios for the period 2017–2030 and for socioeconomic and healthcare variable extrapolated values since 2011 (due to lack of municipal-level data) based on 2000–2010 municipal-level trends, and (ii) the prediction of ACSC mortality rates (the dependent variable) based on the independent variable values and regression model starting from 2010 (using the years before 2017 to calibrate and validate the model). An overview of the methods is provided here, but further details and a theoretical framework are described in Additional file 1, including the modelling calibration process, internal and external validation, parameter distributions for Monte Carlo simulations, and model equations in accordance with the international model reporting guidelines (ISPOR-SMDM) [35].

### Data sources

Two datasets were used for input parameters supplemented with additional data sources. The first contained annual, municipal demographic, socioeconomic, social assistance, and health system variables. Specifically, these were ESF coverage, BFP coverage, illiteracy rate in those aged over 25 years, poverty rate, percentage of population living in urban areas, public healthcare spending (R$100s per person), public hospital beds per 1000 population, private hospital beds per 1000 population, private healthcare insurance coverage, and GDP per capita (R$100s per person). These data, as described elsewhere [17], were obtained from publicly available sources including the Brazilian Ministry of Health (DATASUS) [36] and the Brazilian Institute of Geography and Statistics (IBGE) websites [37, 38]. Socioeconomic variables were forecast from 2011 through exponential decay formulas using historical trends of the previous decade (2000–2010) for each variable in each municipality. The poverty rate was calibrated with national estimates from the National Household Surveys for the period 2011–2014 [39]. Poverty rates for each municipality from 2015 (during the economic recession) were calibrated according to the World Bank national estimates of the most probable scenarios, as described elsewhere [33] (Additional file 1). Annual municipal ESF coverage and BFP coverage values until 2016 were obtained from the Brazilian Ministry of Health’s Department of Primary Care [40] and Brazilian Ministry of Social Development [41]. The data was also supplemented from information on the number of *Mais Médicos* doctors in Brazilian municipalities obtained from the Brazilian Office of the Pan American Health Organization/World Health Organization. The density of *Mais Médicos* doctors in each municipality was used to calculate the programme’s contribution to the municipal ESF coverage (one doctor per 3450 inhabitants as defined by the Brazilian Ministry of Health [42]).

The second dataset contained effect sizes of the associations between changes in these variables and changes in municipal ACSC mortality rates, overall and from the specific group of causes above mentioned, and standardised rate ratios (SRRs) between black and white ACSC mortality. Effect sizes were all obtained from previously published longitudinal fixed effects multiple regression models [17].

### Simulation of the effects on mortality from ACSCs

With all independent variable values from the retrospective and synthetic cohort of municipalities until 2030, and the second dataset of effect sizes, microsimulation forecasting of mortality rates was performed using the same longitudinal fixed effects multiple regression model of the retrospective impact evaluation [17], starting from 2010 in order to calibrate and validate the model. This was undertaken for overall ACSC municipal mortality rates, SRR between black and white ACSC mortality, and ACSC mortality rates from selected groups of ACSCs. Specifically, these were mortality from nutritional deficiencies and anaemia, infectious diseases, and cardiovascular diseases, which were strongly associated with changes in ESF coverage, with a full municipal coverage responsible for mortality reductions of 52%, 21%, and 15% respectively [17]. In addition to generating national trends under each ESF coverage scenario, trends were forecast by poverty quintiles of municipalities. Municipalities were stratified by baseline poverty rates in 2010. For each outcome and each of the four ESF scenarios, 10,000 Monte Carlo simulations were performed, allowing parameters to vary by their assumed underlying distribution—Poisson distribution for the baseline mortality rates (as used in the retrospective study) and normal distributions for all other variables and parameters. For the effect sizes of each variable, the variance of the normal distribution was calibrated with the confidence interval of its effect size parameter (rate ratios) from the reference study [17]. Mean values and credible intervals (CI) (the 2.5% and 97.5% quantiles of the distribution) are reported.

### Calibration and validation of the models

As all model parameters were derived from a retrospective impact evaluation, only calibration of the secular trend for ACSC mortality was necessary (assuming it would have been different from the previous decade). The calculated—and adjusted for undernotification—ACSC mortality rate for 2010–2015 (the most updated available) from the Brazilian Ministry of Health [36] was used to calibrate the coefficient of the time variable within the regression models. Internal validity of the model was assessed by conducting the fixed effects longitudinal regression models employed in the retrospective impact evaluation on the synthetic dataset (years 2017–2030) produced by the microsimulation and verifying that the obtained coefficients were identical to those introduced as inputs in the model. External validation of the model was undertaken using data from the Global Burden of Disease on overall mortality [43] for the period 2010–2015, estimating ACSC mortality rates using this data, and undertaking linear regression of the predicted (from the microsimulation) versus observed (Global Burden of Disease) values. The proportion of variance (R2) explained was also examined, and the observed values were verified to ensure they lay within the simulated 95% CIs. Further detail on model calibration and validation is available in text 1 of Additional file 1. The model was coded and implemented in R v.3.4.0.

### Sensitivity analysis

Multiple sensitivity analyses were undertaken to verify model robustness and assumptions within the ESF scenarios. Firstly, alternative situations of contracting ESF coverage were tested to model the possibility of a greater and lesser impact of federal funding cuts on ESF coverage. Secondly, longer ESF coverage duration effects were explored, and the ESF effect size was also stratified based on poverty rate level of the municipalities. Thirdly, the potential effect from different economic crisis and changes in poverty rate increases were also tested, according to a forecast from a previous study [33], and fourthly, different time trends were evaluated to test the impact of different approaches to modelling secular trends in ACSC mortality. All sensitivity analyses are available in Additional file 1.

## Results

Mean municipal ESF coverage and values of the covariates employed in the models are shown in Table 1. As modelled under scenario 1 (status quo), mean municipal ESF coverage remains constant (at 80.4%) until 2030, whereas under scenarios 2 (contracting ESF coverage due to fiscal austerity) and 3 (contracting ESF coverage and MMP termination), mean coverage falls to 37.8% and 16.0% respectively. Under scenario 4 (UHC), ESF coverage reaches 100% in 2030.

**Table 1:** Mean values and standard deviation of independent municipal-level variables for the years 2015, 2020, and 2030

	Variables	2015	2020	2030
Static ESF coverage (scenario 1)	ESF coverage	84.7% (22.2%)	80.4% (23.5%)	80.4% (23.5%)
Contracting ESF coverage (scenario 2)	ESF coverage	84.7% (22.2%)	66.5% (20.0%)	37.8% (13.9%)
Contracting ESF coverage and *Mais Médicos* termination (scenario 3)	ESF coverage	84.7% (22.2%)	37.0% (30.0%)	16.0% (18.6%)
Universal Health Coverage (scenario 4)	ESF coverage	84.7% (22.2%)	95.6% (4.7%)	99.9% (0.002%)
All scenarios	BFP municipal coverage	33.5% (22.1%)	37.9% (29.1%)	29.7% (25.3%)
	Poverty rate	13.8% (15.3%)	18.4% (21.5%)	12.0% (17.4%)
	Log of illiteracy rate	− 2.37 (0.79)	− 2.98 (1.06)	− 3.38 (1.27)
	Urbanisation rate	68.4% (22.2%)	71.5% (22.4%)	75.8% (23.1%)
	Public hospital beds	0.13 (0.21)	0.12 (0.29)	0.13 (0.25)
	Private hospital beds	0.04 (0.10)	0.05 (0.11)	0.05 (0.12)
	Log of private healthcare insurance	− 5.62 (1.62)	− 5.52 (1.66)	− 5.46 (1.73)
	Log of GDP per capita	− 4.70 (0.71)	− 4.54 (1.02)	− 4.48 (1.46)

Mean values reported and standard deviations shown in parentheses. ESF coverage, BFP coverage, poverty rate, and urbanisation rate expressed as percentages. Private healthcare insurance and GDP per capita are log-transformed. Illiteracy rate is the illiteracy rate of those aged 25 years and over and is log-transformed. GDP is log-transformed. Public and private hospital beds are expressed per 1000 municipal inhabitants

*BFP Bolsa Família* Programme, *ESF Estratégia Saúde da Família* (Family Health Strategy), *GDP* gross domestic product

**Fig. 1:**
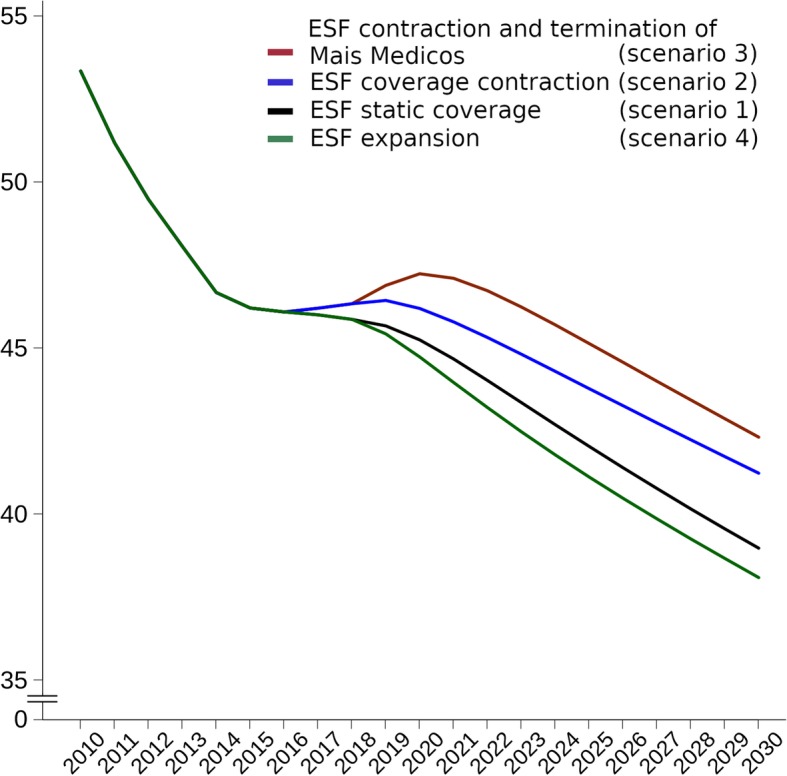
Mean municipal mortality rates from ambulatory care-sensitive conditions (ACSCs) under four ESF scenarios (2010–2030). ACSC mortality expressed per 100,000 inhabitants; the four ESF scenarios are (from highest to lowest ACSC mortality rates in 2030) as follows: ESF contraction and termination of *Mais Médicos* (scenario 3) (red line), ESF contraction (scenario 2) (blue line), static ESF coverage (scenario 1) (grey line), and UHC (scenario 4) (green line). ESF *Estratégia Saúde da Família* (Family Health Strategy)

Under all four scenarios, mean municipal ACSC mortality rates are forecast to continue declining, albeit at different rates (Fig. 1). The reduction of the decline from 2015 is associated with the beginning of the economic crisis. Under the austerity scenarios of either decreasing ESF coverage whilst maintaining the MMP or decreasing ESF coverage with MMP termination, the mean ACSC mortality rates in 2030 would be expected to be 5.80% (95% CI 4.23–7.35%; 27,685 excess premature/under-70 deaths from ACSCs) and 8.60% (95% CI 7.03–10.21%; 48,546 excess premature deaths) higher respectively than when maintaining ESF coverage at current levels. When compared to 100% ESF coverage, mean ACSC mortality will be 11.12% higher (95% CI 9.47–12.76%; 83,937 excess premature deaths) under the full austerity scenario (Table 2).

**Table 2:** Ratios of ACSC mortality rates under ESF scenarios compared to constant ESF coverage in 2020 and 2030

	2020	CI	2030	CI
	Ratio of ACSC mortality rates		Ratio of ACSC mortality rates	
Static ESF coverage (scenario 1)	1 (Ref)		1 (Ref)	
Contracting ESF coverage (scenario 2)	1.021	1.006–1.036	1.058	1.042–1.074
Contracting ESF coverage and *Mais Médicos* termination (scenario 3)	1.044	1.029–1.059	1.086	1.071–1.102
Universal Health Coverage (scenario 4)	0.989	0.974–1.003	0.977	0.963–0.992

*CI* credible intervals, *ESF Estratégia Saúde da Família* (Family Health Strategy), *ACSC* ambulatory care-sensitive condition

**Fig. 2:**
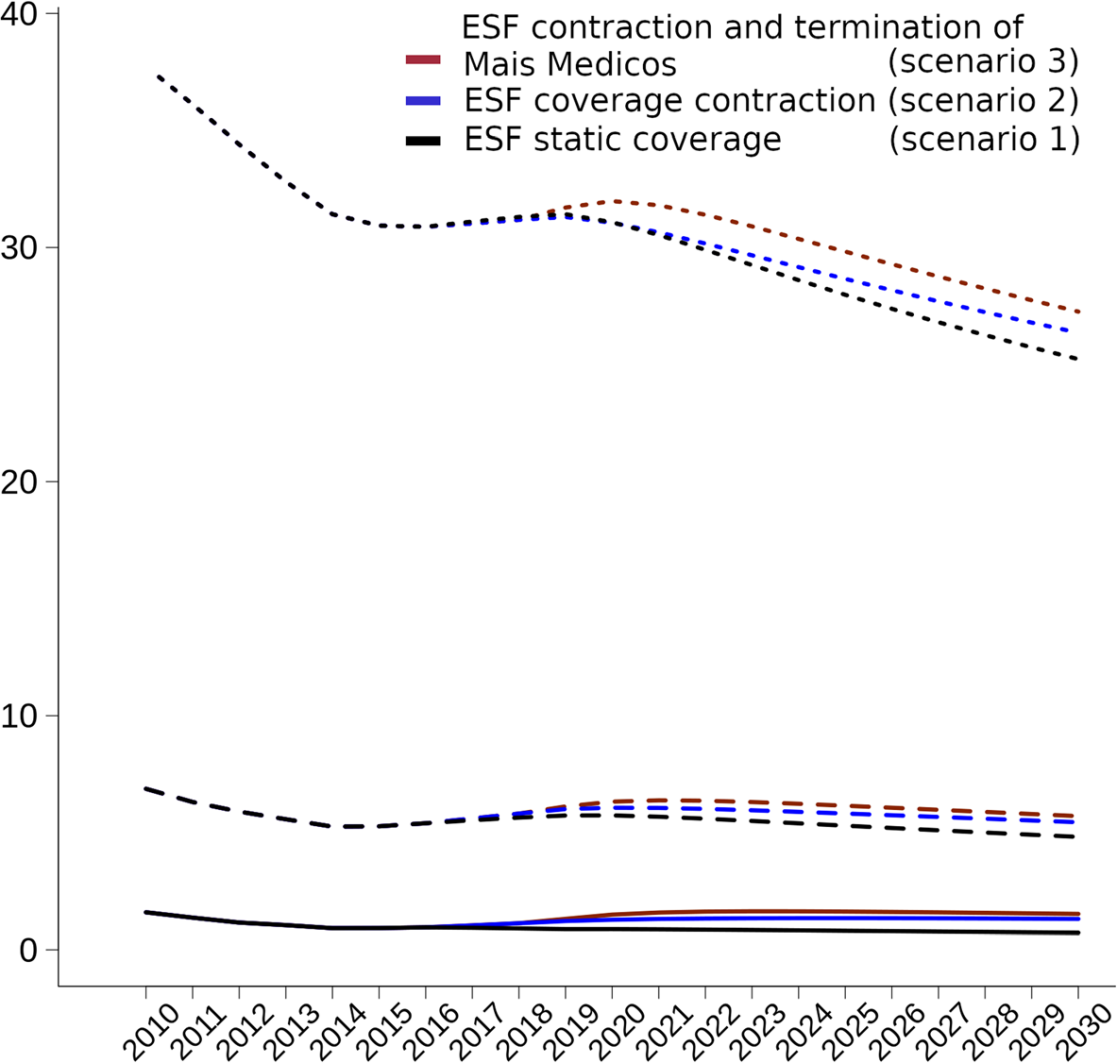
Mean municipal mortality rates for subgroups of ambulatory care-sensitive conditions (ACSCs) under ESF scenarios for 2010–2030: cardiovascular diseases (dotted line), infectious diseases (dashed line), and nutritional deficiencies and anaemia (full line). ACSC mortality expressed per 100,000 inhabitants; the three ESF scenarios are (from highest to lowest ACSC mortality rates in 2030 for all three graphs) as follows: ESF contraction and termination of *Mais Médicos* (scenario 3) (red line), ESF contraction (scenario 2) (blue line), and static ESF coverage (scenario 1) (grey line); ESF *Estratégia Saúde da Família* (Family Health Strategy)

Examining groups of ACSCs reveals divergent trends by scenarios (Fig. 2). Compared to the constant ESF coverage, the ACSC mortality rates in 2030 under decreasing ESF coverage and MMP termination (scenario 3) would be 111.5% (95% CI 101.3–122.1%), 18.4% (95% CI 16.2–20.7%), and 8.30% (95% CI 6.38–10.21%) higher for nutritional deficiencies and anaemia, infectious diseases, and cardiovascular disease, respectively, due to the different ESF effectiveness on these diseases (Additional file 1).

**Fig. 3:**
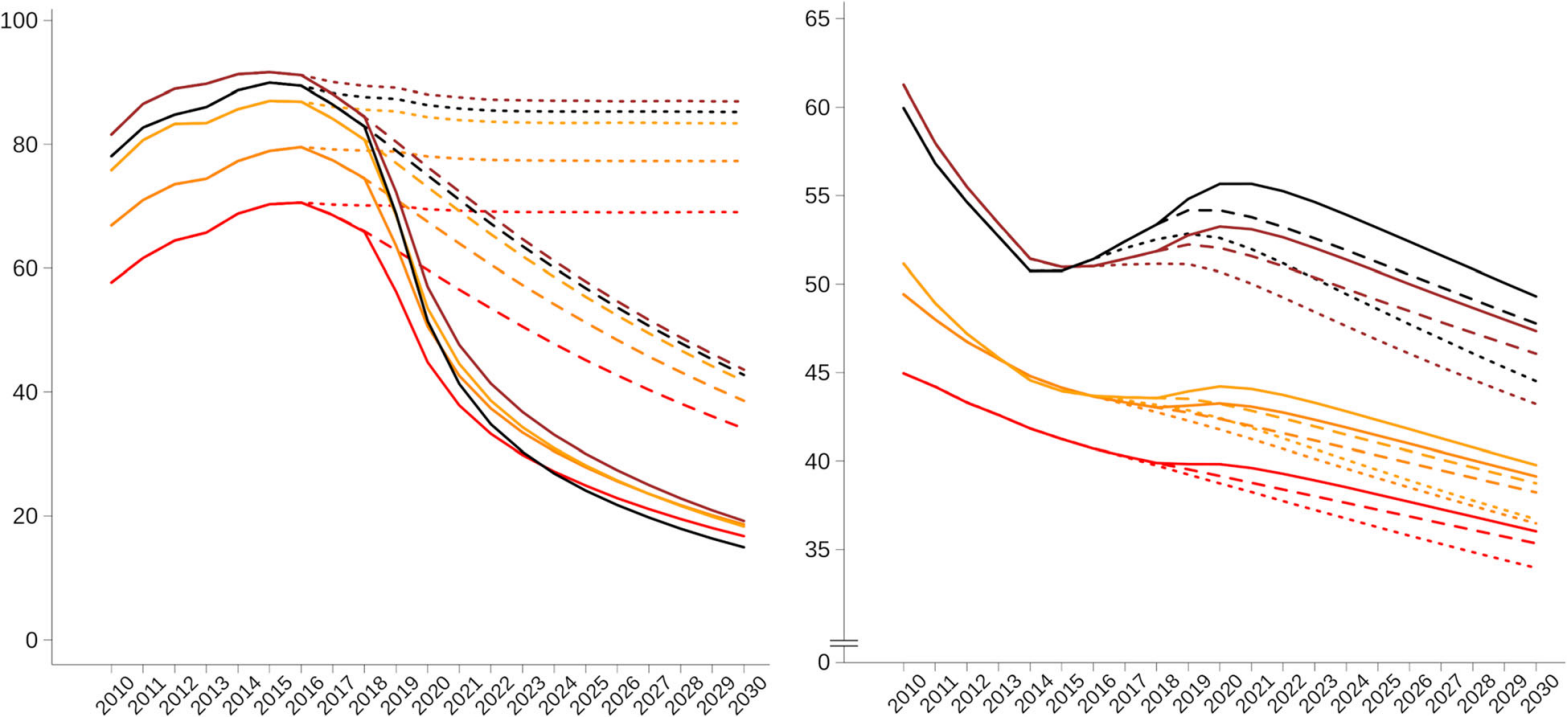
Mean municipal ESF coverage and ACSC mortality rates by poverty quintiles of municipalities under ESF scenarios (2010–2030). ESF coverage expressed as percentage; ACSC mortality expressed per 100,000 inhabitants; the quintiles of municipalities by 2010 poverty rate are (from highest to lowest poverty rates in 2010) as follows: black—first quintile (poorest), brown—second quintile, yellow—third quintile, orange—fourth quintile, and red—fifth quintile (richest); the three ESF scenarios are ESF contraction and termination of *Mais Médicos* (scenario 3) (full line), ESF contraction (scenario 2) (dashed line), and static ESF coverage (scenario 1) (dotted line). ESF *Estratégia Saúde da Família* (Family Health Strategy), ACSC ambulatory care-sensitive condition

Both austerity scenarios would most negatively impact the poorest quintile of municipalities (Table 3 and Fig. 3). In the poorest quintile of municipalities under contracting ESF coverage and MMP termination (scenario 3), in terms of the rate difference, there would be 4.79 (95% CI 3.32–6.26) higher ACSC mortality rate than under static ESF coverage (scenario 1), whilst in the richest quintile, this difference will be 2.04 (95% CI 0.80–3.30) (Table 3). This would result in a ACSC mortality concentration index—based on municipality poverty rates—11.77% higher (95% CI 0.31–22.32%) than under constant ESF coverage.

**Table 3:** Differences in ACSC mortality rates between contracting ESF scenarios and static ESF coverage by municipality poverty quintile for 2015–2030

	Contracting ESF coverage (scenario 2)		Contracting ESF coverage and *Mais Médicos* termination (scenario 3)	
	RD	CI	RD	CI
Q1 (poorest)	3.26	1.83–4.67	4.79	3.32–6.26
Q2	2.84	1.42–4.27	4.12	2.68–5.53
Q3	2.02	0.77–3.29	3.04	1.80–4.31
Q4	1.73	0.50–2.98	2.62	1.37–3.86
Q5 (richest)	1.37	0.78–2.62	2.04	0.80–3.30

Differences in ACSC mortality expressed per 100,000 inhabitants; rate difference between ESF scenario 1 (static ESF coverage)

*RD* rate difference, *CI* credible interval, *ESF Estratégia Saúde da Família* (Family Health Strategy)

**Fig. 4:**
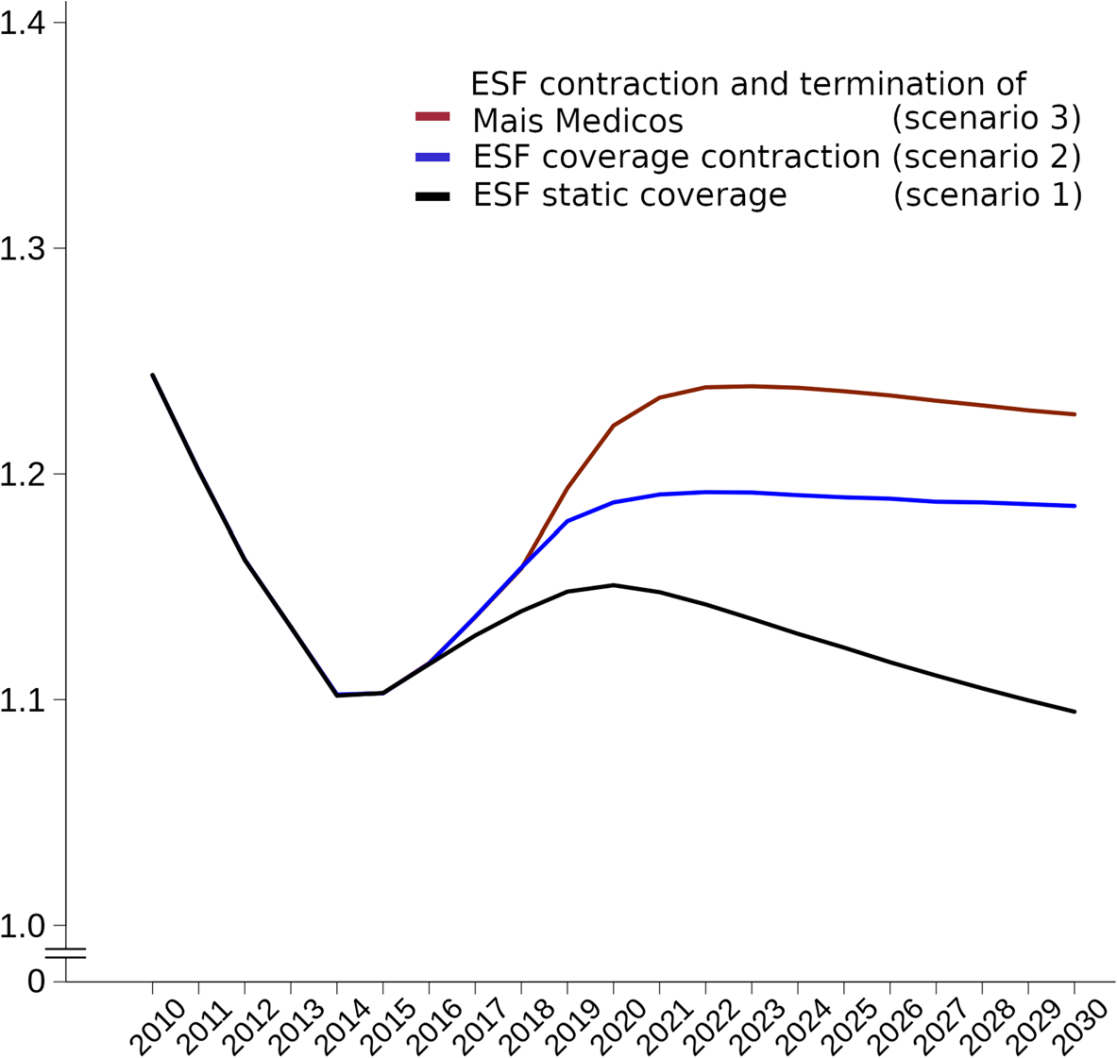
Standardised rate ratios (SRRs) for ACSC mortality from between black/pardo and white populations under ESF scenarios for 2010–2030. Standardised rate ratios (SRRs) of ACSC mortality is black/pardo ACSC mortality divided by white ACSC mortality; the three ESF scenarios are ESF contraction and termination of *Mais Médicos* (scenario 3) (red line), ESF contraction (scenario 2) (blue line), and static ESF coverage (scenario 1) (grey line). ESF *Estratégia Saúde da Família* (Family Health Strategy), ACSC ambulatory care-sensitive condition

Examining inequalities in black/pardo and white ACSC mortality reveals that racial group inequalities would increase under both austerity scenarios compared with constant ESF coverage (Fig. 4). The standardised rate ratios (SRRs) of ACSC mortality rate between black/pardo and white ACSC mortality would be 8.36% (95% CI 3.16–13.70%) and 12.08% (95% CI 6.80–17.54%) higher in 2030 under scenarios 2 and 3 respectively.

### Sensitivity analysis

Findings from our sensitivity analyses support the robustness of the findings (Additional file 1). Firstly, varying the magnitude of declines in municipal ESF coverage associated with fiscal austerity measures reveals that there is a dose-response relationship (larger ESF coverage reductions are associated with higher ACSC mortality), consistent with the main analyses. Introducing different duration effects of the ESF coverage produced similar results, as did varying the effectiveness of ESF in reducing ACSC mortality between municipalities with different levels of poverty. Modelling different economic recessions and changes in poverty rates over the coming years yielded highly similar results both in aggregate and inequality analyses. Introducing different secular trends in ACSC mortality rates alters the general trends over time, but the relative differences between scenarios (in terms of the rate ratios) remain unchanged.

## Discussion

Under austerity scenarios of decreasing ESF coverage with and without MMP termination, ACSC premature mortality rates would be expected to be 8.6% and 5.8% higher respectively in 2030 compared with maintaining the current ESF coverage. Excess premature deaths from ACSCs in the first—most probable—scenario will be almost 50,000 between 2017 and 2030. Compared with achieving 100% ESF coverage (UHC scenario), mortality rates would be 11.1% higher in 2030 if the ESF contracts and the MMP ends, corresponding to almost 85,000 premature deaths. Reductions in ESF coverage under austerity scenarios would disproportionately impact the poorest municipalities. Existing trends of reducing inequalities in mortality between richest and poorest municipalities and white and black/pardo Brazilians would end with ESF coverage reductions.

The avoidable death estimates of the study should be considered an underestimation of the overall mortality impact of ESF coverage reductions, because they include only under-70 deaths (which represent around 55% of the total Brazilian deaths), and they evaluate only deaths from ACSC (around 13% of the premature deaths), whilst ESF should have smaller effects also on othermortality causes [15].

To our knowledge, this is the first study to forecast the impact of austerity measures on PHC coverage and health outcomes in a middle-income country. Our findings are consistent with the evidence, including studies from MICs, that show investments and pro-equity implementation of PHC can improve health and reduce inequalities [5, 6, 44]—including greater mortality reductions in low-income and black populations [17, 45, 46]. In Europe, in response to the 2008 Great Recession, many countries reduced coverage of public health care—including introducing user charges, reducing services covered, or restricting populations covered [47, 48]. Cuts in public health spending occurred in many European countries under austerity measures, most notably in Greece, Ireland, Latvia, and Portugal [49], which were associated increases in self-reported unmet medical need [50, 51]. Many European countries additionally introduced cost-sharing policies sharply increasing out-of-pocket expenditure on medications and visits to primary care services [52]. These policies are likely to have an adverse impact on health outcomes given that cost has been shown to be an important barrier to optimal medication use and primary care clinic attendance in European settings [53, 54].

There are multiple strengths to this study. We developed a synthetic 14-year cohort of 5507 municipalities based on routinely collected data widely used in previous impact evaluation studies in Brazil [16, 17]. The permitted creation of a correlation structure between variables and modelling of municipality-specific parameters and variable trends based on real data, which have been calibrated with the national-level data. The external validation of the models—using a different data source than the one used for the calibration process—is another strength of the study, together with the extensive sensitivity analyses employed. Our estimates of PHC impacts on mortality are consistent with numerous previous Brazilian studies [15–17, 55, 56]—strengthening forecast theoretical, and modelled, relationships.

One limitation of the study is the assumption that estimates of ESF coverage will decline in proportion to federal funding cuts. The elasticity of ESF coverage in response to federal transfers is assumed in this study to be directly proportional given the heavy reliance of municipalities on federal budgets for health, but shows in sensitivity analyses that varying this relationship does not alter the differences between ESF scenarios substantially. We used mortality from ACSCs as our outcome measure, rather than healthcare-amenable mortality [57], to focus on conditions defined as amenable to PHC within the Brazilian context, to exclude conditions strongly influenced by hospital-based care, to increase the accuracy and reliability of the models, and to be in line with retrospective studies [16, 17]. Despite the fact that, considering the most recent studies [14–17]—ESF has shown its strongest effects on ACSC, we are probably underestimating the overall effects of ESF coverage reductions on the health of the population by selecting only this group of causes. Another limitation of the study is the assumption that ESF units with MMP physicians have the same effectiveness as ESF units in general. This assumption is informed by the fact that MMP physicians are working in similar ESF units, with the same supporting instruments and infrastructure. They are also working to the same clinical guidance for ACS conditions.

The ESF is an internationally recognised model of PHC which aspires to deliver key features of Alma-Ata vision—restated in the draft 2018 Astana declaration (Alma-Ata 2.0) [3, 58]—including proactive and continuous community-based care which is geographically accessible and low cost. Despite facing severe financing and human resource constraints, the ESF has delivered substantial health benefits to the Brazilian population [11, 14]. Brazil’s programme of long-term austerity measures will substantially reduce ESF coverage and associated health benefits especially among the country’s poor—undermining progress towards achievement of SDGs 3 and 10, including achievement of UHC. In this sense, the health and equity impacts of austerity policies in Brazil should be closely monitored to optimise policy learning globally.

## Conclusions

Our findings suggest that austerity measures affecting PHC could contribute to a large number of avoidable deaths and may preclude achievement of SDGs for health and inequality in Brazil and in other low- and middle-income countries.

## Additional file

Additional file 1: Detail on model calibration and validation and findings from the sensitivity analyses. (DOCX 803 kb)

## Abbreviations

ACSC: Ambulatory Care-Sensitive Conditions
BFP: Bolsa Familia Programme
ESF: Estratégia de Saúde da Família
MMP: Mais Médicos programme
PHC: Primary Health Care
SDG: Sustainable Development Goals
UHC: Universal Health Coverage
