# Supplementary material for: Mortality associated with alternative primary healthcare policies: a nationwide microsimulation modelling study in Brazil
Source: BMC Med. 2019 Apr 26;17:82. doi: 10.1186/s12916-019-1316-7 (PMC6485171; doi:10.1186/s12916-019-1316-7)
Supplement: Supplementary file 1 — Detail on model calibration and validation and findings from the sensitivity analyses. (DOCX 803 kb) [file 12916_2019_1316_MOESM1_ESM.docx]

**Supplementary Appendix**

**Mortality associated with alternative primary health care policies: a nationwide microsimulation modelling study in Brazil**

D Rasella; T Hone; LE de Souza; R Tasca; S Basu; C Millett

**1. DETAILED DESCRIPTION OF THE MODELING PROCESS**

The following section provides details of each of these processes in accordance with standard international modelling reporting guidelines (ISPOR-SMDM). The modelling approach was based on a previous national microsimulation study^1^ and was developed based on two stages.

In the first stage, a synthetic cohort of all Brazilian municipalities for the period 2010-2030 was created as an extension of a longitudinal dataset for 2000-2016 used in previous ex-post impact evaluations on PHC performed by the authors^2,3^, where fixed effects multivariate regressions, adjusted for demographic and socioeconomic factors, were used to estimate the effectiveness of the *Estratégia Saúde da Família* (ESF; Family Health Strategy) on mortality from Ambulatory Care Sensitive Conditions (ACSC). Trends in municipal poverty rates and the other demographic and socioeconomic variables were forecast for the years 2017-2030, and ESF coverage according to the ESF scenarios outlined in the manuscript.

In the second stage, for each year, municipality and ESF coverage scenario, ACSC mortality rates (deaths per 100,000 population aged under 70 years of age) were estimated as outcome of the same multivariate fixed effects regressions using the forecast demographic and socioeconomic variables, and ESF coverage (as per the scenario) as input values. Mean ACSC mortality rates were calculated for the whole country and for subgroups of municipalities.

**Purpose of the model and its applications**

The developed model - derived from a core model already used in a recent study on the effects of economic crisis on child mortality^1^ - aims to simulate the effects of socioeconomic and policy coverage changes on health outcomes in Brazil using ecologic-level data and - when available - retrospective ecologic datasets. Flexibility has been introduced in the code to allow simulation of different sets of variables and different regression models.

**Theoretical framework of the model**

The ESF is a community-based model of primary healthcare, centered on family health teams staffed by a doctor, nurse, nurse assistant, and community health workers providing healthcare to locally defined populations. Approximately 3,500 individuals are registered per team and receive a broad package of primary care services including basic curative care, health promotion, health education, and specific targeted programmes such as women and children’s health, HIV/AIDS, infectious diseases, and cardiovascular health. A sizeable evidence base has grown demonstrating the impact of the ESF including on child health. High municipal ESF coverage was associated a 12% reduction in U5MRs over the period 2004-2009,^4^ in addition to studies showing ESF expansion was associated with declines in hospitalisations and mortality from amenable causes.^1,2,5,6^ As a primary healthcare service, there are multiple ways through which the ESF can bring about improvements in health outcomes. ESF teams are responsible for registering and delivering healthcare services to defined local populations. The range of services should be comprehensive and are care free of charge. Firstly, expanded access to curative services may impart improvements in health outcomes for the population. Child and maternal health services are identified as an essential service that must be delivered by ESF teams. Many causes of child deaths (e.g. intestinal infections or vaccine preventable conditions) may be easily preventable by access to basic services. Secondly, community health workers may play an important role in expanding access to health services through outreach and educating mothers and families about potential services and ways of access. Thirdly, health promotion, prevention and educational activities provided by ESF teams may have a more indirect mechanism of action in improving health literacy of enrolled population, preventing uptake of riskier behaviors and exacerbation of existing risk factors and encouraging healthier behaviors.

In the regression model used for the retrospective and forecast evaluation the independent variable were socioeconomic variables: illiteracy rate in those aged over 25 years; poverty rate; percentage of population living in urban areas, GDP per capita and healthcare-related variables: number of public hospital beds per 1,000 inhabitants, number of private hospital beds per 1,000 inhabitants, percentage of inhabitants with private healthcare insurance. Bolsa Familia Program, the largest intervention of poverty-relief in the country, was included as adjusting variable because while not all the mortality causes in the ACSC list are associated and sensitive to poverty, some of them such as Tuberculosis and Intestinal Infectious diseases - among others – are related strongly to the low income of the households.

**Data sources, inputs, outputs, and other parameters**

Two types of input data were introduced as parameters in the models: the first were municipality-specific demographic and socioeconomic variables values-including their municipality-specific trends plus BFP and ESF coverage values; the second were the effect sizes of all the regression independent variables on ACSC mortality, overall and from specific causes.

Values for socioeconomic variables (illiteracy rate in those aged over 25 years; poverty rate; percentage of population living in urban areas) obtained from the Brazilian National Census data of 2010 (IBGE 2017) for the years 2011-2030 were extrapolated. Poverty rate trends were calibrated with national trends from the National Household Surveys^3^ for the period 2011-2014. BFP coverage values were obtained -for the period 2010-2016- from the Ministry of Social Development. Forecast (2017-2030) BFP coverage for each municipality was simulated according to declines in poverty rates as described elsewhere^1^. For the other variables (number of public hospital beds per 1,000 inhabitants, number of private hospital beds per 1,000 inhabitants, percentage of inhabitants with private healthcare insurance, GDP per capita) real values were used for the year 2010, and after extrapolated. Trends of GDP per capita for the period 2010-2017 were based in official national-level GDP per capita estimates and for the 2018-2030 in government projections.^7^ As tested in the sensitivity analyses, being these variables and their trends common in all ESF scenarios, any alternative forecast was not changing the findings of the study, expressed as rate ratios between scenarios. For each variable the municipality-specific extrapolation was performed through exponential decay formulas using the municipality-specific time trends obtained from the retrospective dataset in 2000-2010. Apart from the trends of poverty rate and GDP affected by economic crisis - modeled and calibrated using previous simulations from the World Bank as described elsewhere^1^ , the trends of the other determinants of ACSC MRs has been modelled as not affected by the economic crisis and their specific trends where obtained - for each municipality - from the linear trend of the decade 2000-2010 expressed as percent decrease for the year 2010 an inserted in the percent decrease/exponential decay formula:

*V_it_ = V_i_ (1-P_i_)^Kt^*

*V_it =_* Value of the variable for the municipality i at the time t

*V_i =_* Value of the variable for the municipality i at the time 0 (year 2010).

*t* = Time

*P_i_* _=_ Percent decrease of the municipality i (obtained for the period 2000-2010)

*K* = Calibration term

For each municipality and each variable trends for the period 2010-2030 have been estimated according to the above formula, and poverty rate- the most influential in the model - has been calibrated varying the term K with the real variable values – when data were available - at the country level for the years 2011-2014 obtained from the National Household Surveys (PNAD).^3^

**ESF coverage**

For the main exposure variable, ESF coverage, values were obtained -for the period 2010-2016 from the Ministry of Health’s Department of Primary Care.^8^ Forecast (2017-2030) ESF coverage data points for each municipality were simulated according to the different scenarios defined in Figure A.

***Figure A - Box plots of municipal ESF coverage by ESF scenarios for the period 2010-2030.***

**
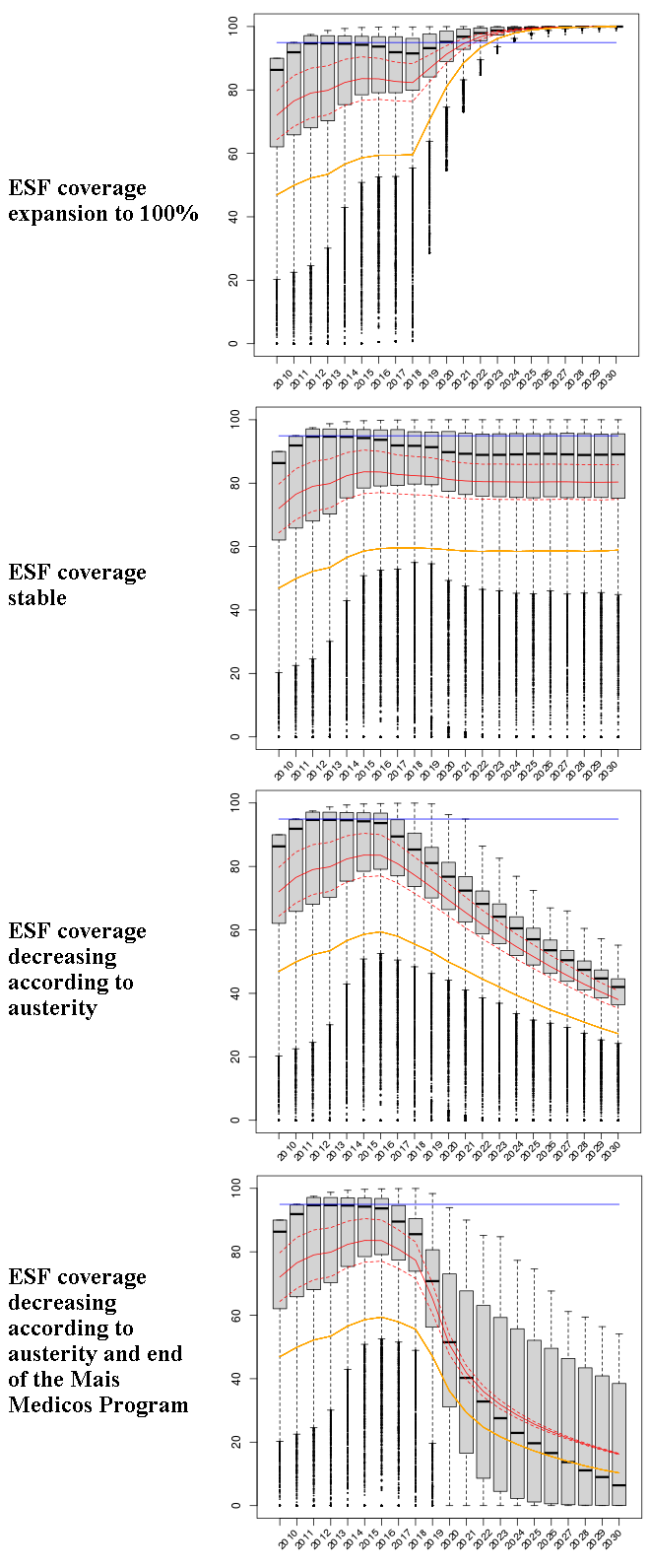

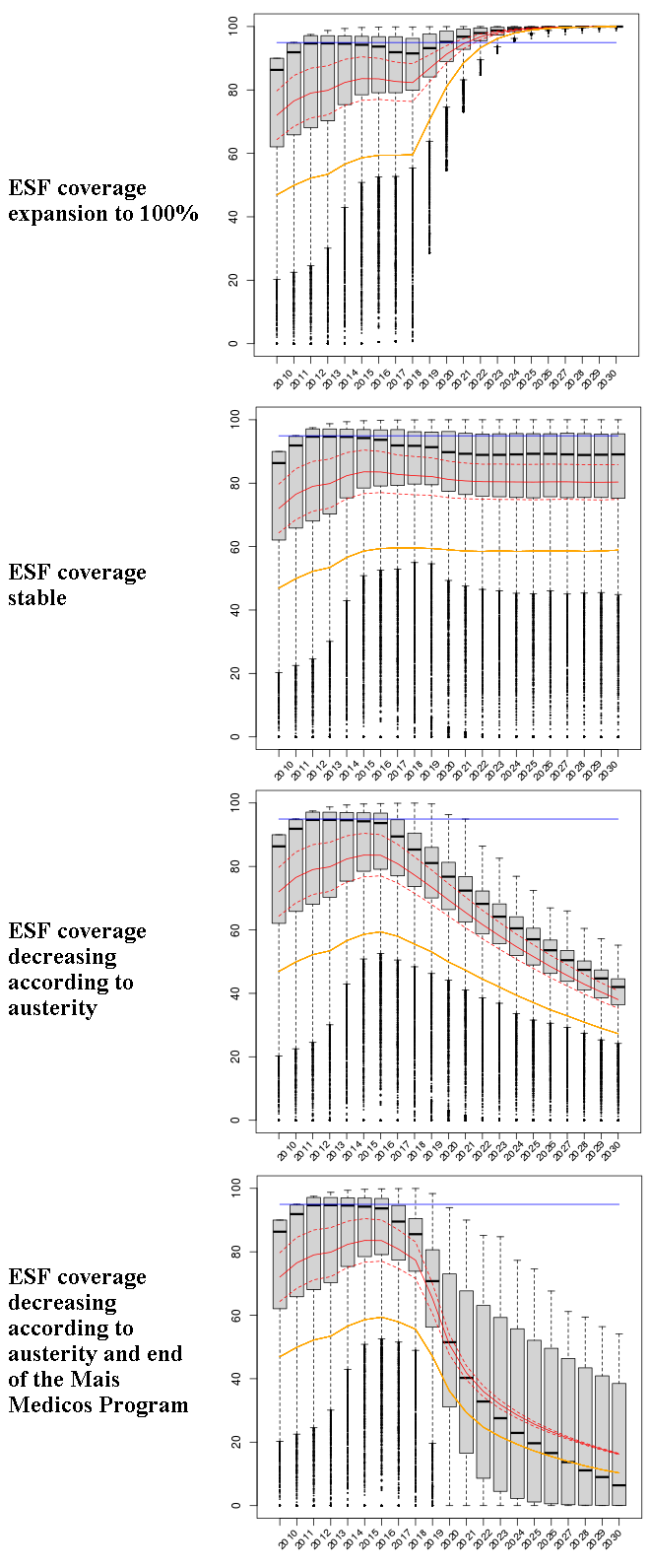
**

**Effect size parameters in the model**

The effect sizes - expressed in terms of rate ratios - of each continuous variable for each specific outcome are obtained from the retrospective impact evaluation^2^ (Table A).

***Table A - Rate ratios in ACSC mortality rates for dependent variables***

|  | **Rate Ratios for Under 70 ACSC mortality rates** | | | |
| --- | --- | --- | --- | --- |
|  | **Overall** | **Anemia and nutritional deficiencies** | **Infectious diseases** | **Cardiovascular diseases** |
| **ESF coverage** | 0.89 (0.84 -0.93) | 0.48 (0.23 -0.95) | 0.79 (0.53 -1.19) | 0.85 (0.80 -0.90) |
| **BFP coverage** | 0.94 (0.85 -1.04) | 0.11 (0.02 -0.51) | 0.81 (0.64 -1.02) | 1.16 (1.01 -1.34) |
| **Poverty Rate** | 1.81 (1.25 -2.61) | 11.26 (0.14 -849.71) | 4.99 (1.49 -16.68) | 2.51 (1.50 -4.24) |
| **Log of Illiteracy Rate** | 0.85 (0.73 -0.99) | 2.73 (0.34 -21.70) | 0.40 (0.21 -0.74) | 0.99 (0.85 -1.15) |
| **Urbanisation rate** | 0.66 (0.43 -1.02) | 0.0004 (0.0001 -2.12) | 0.04 (0.004 -0.46) | 0.98 (0.56 -1.70) |
| **Public hospital beds** | 1.01 (0.95 -1.07) | 1.22 (0.61 -2.45) | 1.15 (0.42 -1.95) | 1.03 (0.96 -1.09) |
| **Private hospital beds** | 1.10 (0.90 -1.33) | 0.39 (0.02 -9.06) | 1.67 (0.60 -4.68) | 0.97 (0.78 -1.21) |
| **Log of Private healthcare insurance** | 0.92 (0.84 -1.01) | 0.41 (0.14 -1.22) | 1.35 (0.95 -1.93) | 0.91 (0.82 -1.01) |
| **Log of GDP** | 0.89 (0.81 -0.98) | 0.29 (0.29 -0.86) | 1.09 (0.77 -1.56) | 0.94 (0.83 -1.07) |

*Rate ratios are the ratio of mortality rates for a one unit increase in the dependent variable. BFP - Bolsa Família Programme; ESF - Estratégia Saúde da Família (Family Health Strategy); GDP – Gross Domestic Product; ESF coverage, BFP coverage, poverty rate, and urbanisation rate expressed as percentages. Private healthcare insurance is also expressed as a percentage, but is log-transformed. Illiteracy rate is the illiteracy rate of those aged 25 y and over and is log-transformed. Public healthcare spending is expressed as hundreds of Brazilian reais per person, as is GDP, although GDP is log-transformed. Public and private hospital beds are expressed per 1,000 municipal inhabitants.*

The different effect sizes of the variables, mainly ESF coverage, explain the different behaviors of the curves, and the final different RR between scenarios: compared to constant ESF coverage, the ACSC mortality rates in 2030 under decreasing ESF coverage and MMP termination (scenario 3) would be 111.5% (95%CI:101.3%-122.1%), 18.4% (95%CI:16.2%-20.7%), and 8.30% (95%CI: 6.38%-10.21%) higher for nutritional deficiencies and anemia, infectious diseases, and cardiovascular disease respectively (Figure B).

***Figure B - Mean municipal mortality rates for subgroups of ambulatory care-sensitive conditions (ACSCs) under ESF scenarios for 2010-2030.***

***a. Nutritional deficiencies and anemia b. Infectious Diseases c. Cardiovascular diseases***

***
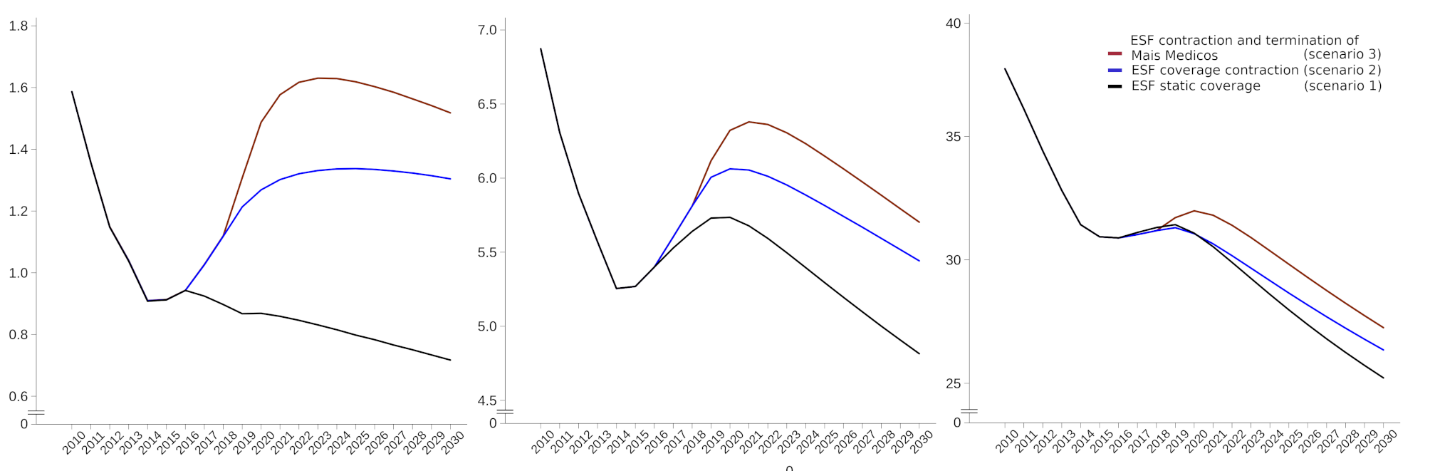
***

*ACSC mortality expressed per 100,000 inhabitants; the three ESF scenarios are (from highest to lowest ACSC mortality rates in 2030 for all three graphs): ESF contraction and termination of Mais Médicos (scenario 3) (red line); ESF contraction (scenario 2) (blue line); and static ESF coverage (scenario 1) (grey line); ESF - Estratégia Saúde da Família (Family Health Strategy);*

**Parameters distributions of the Monte-Carlo simulations**

For each Monte Carlo simulation the effect size parameters were varied according to a normal distribution with the 95% of the values included in the 95%CIs of Table A. Other parameters varied for each Monte Carlo simulation were the trend values for each variable in each municipality, used -as explained above in the exponential decay formula- to forecast the values of the variables (for each municipality) up to 2030: a normal distribution with as mean the historical trend of the previous decade (2000-2010) and as coefficient of variation 10%. Other parameters varied in the Monte Carlos simulations and their distributional characteristics are shown in Table B.

***Table B – Parameters varied in the Monte Carlos simulations and their distributional characteristics***

|  | **Distribution** | **Mean** | **Coefficient of Variation (%)** |
| --- | --- | --- | --- |
| **Municipal-level trends for each variable (percent changes)** | Normal | Historical trend of the previous decade (2000-2010) | 10.0 |
| **Effect size of the all independent variables** | Normal | According to Table A | According to Table A |
| **Effect size of the time variable** | Normal | 0.012 | 10.0 |
| **Municipal-level trends for population under 70 (percent changes)** | Normal | Historical trend of the previous decade (2000-2010) | 1.0 |

**Model equation**

Panel data post-sample forecasting is a type of forecasting which uses the time-series dimension of the dataset and forecasts the values of variables, both dependent and independent, from existing observations.^9,10^ Fixed effects negative-binomial longitudinal (panel) regression models and their parameters obtained from ex-post impact evaluations were used to perform a post-sample forecasting of ACSC mortality rate.

Each outcome, for a specific year and specific municipality, was estimated as the product of the FE term of the municipality, the independent variables with their effects expressed as rate ratios (RR), and the dummy time variable with its RR, according to the following equation:

*Log* (*ACSCMR_it_)= α_i_ + β_1_ BFP _it_+ β_2_ ESF _it_+ β_k_ X_k it_ + β_3_t*

Where:

*t* refers to the year and *i* refers to an individual municipality,

*ACSCMR_it_* is the U5MR in municipality *i* in year *t*,

*BFP _it_* is the coverage of BFP in municipality *i* in year *t* with a coefficient of *β_1_,*

*ESF _it_* is the coverage of ESF in municipality *i* in year *t* with a coefficient of *β_2_,*

*X_kit_* refers to each covariate (*k)* for municipality *i* in year *t* with coefficient *β_k_,*

*t* is the time dummy variable with coefficient *β_3_,*

*α_i_* is the fixed effect (time-invariant) term for each municipality.

Time was included in the model to capture omitted variables which could contribute to trends in ACSC mortality rates affecting all municipalities.

**Calibration of the models**

As explained above, all parameters of the models were derived from a pre-existing retrospective dataset and ex-post impact evaluation.^2^ The only calibrated variable of the model was the effect size of the time variable, representing secular trends in ACSC mortality rates not captured by the adjusting variables of the regression and assumed to be different from the previous decade. The calibration was performed through comparison of the linear trend of ACSC mortality rates calculated from the death notifications of the Mortality Information System (*Sistema de Informação sobre Mortalidade (*SIM)), in a subset of 1,622 municipalities with adequate vital statistics reporting used in previous studies ^1,2^ for the period 2000-2015 (2015 was the last year available).

This time trend was compared with the ACSC mortality rate time trend produced by the microsimulation models from the same municipalities for the period 2010-2015, and the coefficient of the time variable of the model was calibrated in order to forecast ACSC mortality rates with the closest time trend to the real data. The time factor in the microsimulation model was finally calibrated as an annual reduction of 1.2% in the ACSC mortality rate.

**Internal validation of the model**

Internal validity of the model was assessed fitting the fixed effects negative binomial multivariate regression described above - and used for the microsimulation - on the synthetic dataset created for the period 2010-2030, and verifying that the obtained coefficients for the main variables were the same than the ones introduced as inputs in the model (and derived from the retrospective impact evaluation).

**External validation of the Models**

The external validation of the model was undertaken comparing the overall national ACSC mortality rate forecast by the microsimulation model calculated from the death notifications of the Mortality Information System (*Sistema de Informação sobre Mortalidade (*SIM)) with ACSC mortality rate estimates produced using the IHME -GBD dataset (http://www.healthdata.org/gbd/data) of age-standardized mortality rates from all causes for all Brazil, adjusted for under-notification.

An estimation of the proportion of age-standardized ACSC MR - calculated from the death notifications of the Mortality Information System (*Sistema de Informação sobre Mortalidade (*SIM)) - in the total age-standardized mortality of the country – obtained from GDB estimates - was undertaken along the period 2000-2015 (2015 was the last year available). The yearly proportions obtained were fitted with a linear regression along the period and yearly predicted values from the regression were corrected with an empirically estimated under-notification factor, obtained as the difference between the ACSC MR produced by the model – which corrects for under-notification - in 2010 with the predicted by the linear regression – theoretically vulnerable to under-notification. The estimates of under-notification-adjusted ACSC MR for the years 2011-2015 were produced multiplying the obtained proportions for 2011-2015 with the age-standardized mortality rates from GBD estimates for the same period.

Figure C shows the estimated linear regression and the correlation coefficients (R^2^) of predicted values by the model vs observed (estimated as described above). Additionally, it was verified that all points of ACSC MR estimates where included in the 95% Confidence Intervals of our simulation.

***Figure C - Linear regression and correlation coefficient (R^2^) of predicted vs observed values, and trend of the simulated overall ACSC mortality rate with CIs vs the ACSC mortality rate estimated from the IHME GBD for Brazil (2010-2015).***


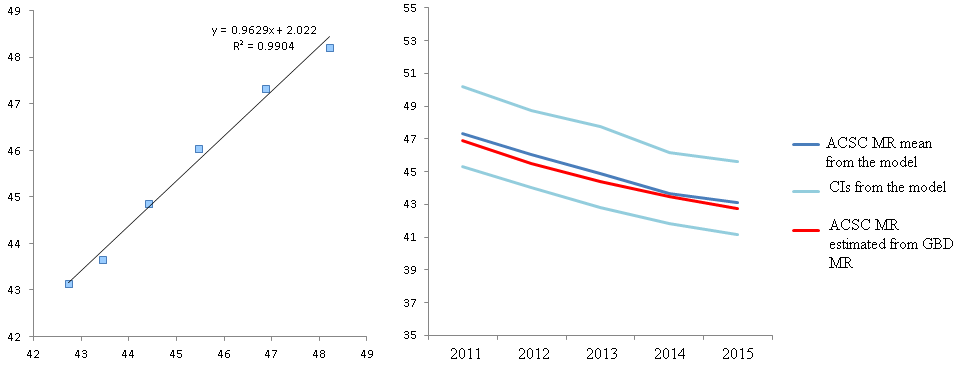


National geographical patterns, obtained projecting the municipality variables on the country map, and correlation structures of all the variables of the model at the beginning and at the end of the simulation, have been used as robustness analysis to verify if important and non-expected variations were introduced by the simulation. Brazil is characterized by specific areas of poverty and socioeconomic vulnerability, which correspond closely to several health outcome patterns. Figure D shows that geospatial patterns will remain considerably similar even with changes in the values of the variables between 2010 and 2030.

***Figure D - Geospatial patterns of municipal ACSC mortality rates in the first (2010) and last year (2030) of the simulation.***


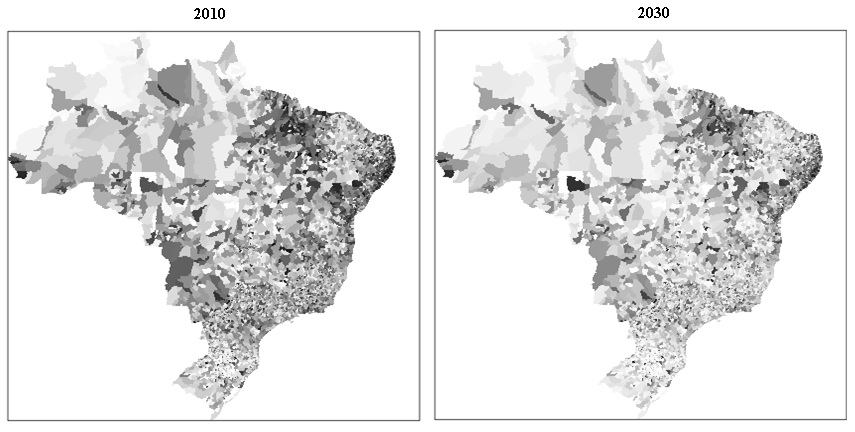


*ACSC mortality expressed per 100,000 inhabitants; ACSC - ambulatory care-sensitive conditions;*

**Summary of Results (more broadly explained in the manuscript)**

As modelled under scenario one (status quo), mean municipal ESF coverage remains constant (at 80.4%) until 2030, whereas under scenarios two (contracting ESF coverage due to fiscal austerity) and three (contracting ESF coverage and MMP termination) mean coverage falls to 37.8% and 16.0% respectively. Under scenario four (UHC), ESF coverage reaches 100% in 2030.

Under the austerity scenarios of either decreasing ESF coverage whilst maintaining the MMP, or decreasing ESF coverage with MMP termination, the mean ACSC mortality rates in 2030 would be expected to be 5.80% (95%CI: 4.23%-7.35%; 27,685 excess under 70 deaths from ACSCs) and 8.60% (95%CI: 7.03%-10.21%; 48,546 excess deaths) higher respectively than when maintaining ESF coverage at current levels. When compared to 100% ESF coverage, mean ACSC mortality will be 11.12% higher (95%CI: 9.47%-12.76%; 83,937 excess deaths) higher under the full austerity scenario.

Compared to constant ESF coverage, the ACSC mortality rates in 2030 under decreasing ESF coverage and MMP termination (scenario 3) would be 111.5% (95%CI:101.3%-122.1%), 18.4% (95%CI:16.2%-20.7%), and 8.30% (95%CI: 6.38%-10.21%) higher for nutritional deficiencies and anaemia, infectious diseases, and cardiovascular disease respectively.

In the poorest quintile of municipalities under contracting ESF coverage and MMP termination (scenario 3), in terms of the rate difference, there would be 4.79 (95%CI:3.32-6.26) higher ACSC mortality rate than under static ESF coverage (scenario 1), whilst in the richest quintile this difference will be 2.04 (95%CI:0.80-3.30). This would result in a concentration index by municipality poverty 11.77% higher (95%CI:0.31%-22.32%) higher than under constant ESF coverage.

The standardised rate ratios (SRR) of ACSC mortality rate between black/pardo and white ACSC mortality would be 8.36% (95%CI:3.16%-13.70%) and 12.08% (95%CI:6.80%-17.54%) higher in 2030 under scenarios 2 and 3 respectively.

**Sensitivity analysis**

a. Varying policy response intensities

As sensitivity analysis, we modelled additional possibilities that ESF coverage declines at different intensities in relation to federal austerity measures (Table C). Under all these scenarios austerity-induced contractions in ESF coverage were associated with excessive under-70 deaths from ACSC compared to the scenario of static ESF coverage.

|  | **Yearly ESF percent reduction** | | | |
| --- | --- | --- | --- | --- |
|  | **2%** | **4%** | **5.50%** | **7%** |
| **Static ESF coverage (scenario 1)** | 1 (ref) | 1(ref) | 1(ref) | 1(ref) |
| **Contracting ESF coverage (scenario 2)** | 1.028 | 1.047 | 1.057 | 1.067 |
|  | (1.013-1.043) | (1.031-1.063) | (1.042-1.073) | (1.051-1.083) |
| **Contracting ESF coverage and *Mais Médicos* termination (scenario 3)** | 1.064 | 1.078 | 1.086 | 1.092 |
|  | (1.047-1.079) | (1.061-1.094) | (1.069-1.102) | (1.074-1.107) |

***Table C - ACSC mortality rate ratios between the ESF coverage scenarios and static ESF coverage for 2030 with varying intensities of ESF coverage reduction***

*SF - Estratégia Saúde da Família (Family Health Strategy); ACSC – ambulatory care sensitive condition*

b. Varying secular trends in ACSC mortality rates

In order to verify that an inaccurate calibration of the time coefficient or that unexpected secular trends would not change the findings of the study, models have been run with the same inputs and parameters but different time trend coefficients – in terms of annual reductions in ACSC mortality rates. The results in terms of effects of austerity-induced ESF contraction were identical to main results (Table D).

***Table D – Rate ratios in ACSC mortality rates between static ESF coverage (scenario 1) and contracting ESF coverage and Mais Médicos termination (scenario 3) for the year 2030 with different secular trends***

| **Coefficient of time variable** | **Rate Ratio** | **Credible Intervals** |
| --- | --- | --- |
| 0.010 | 1.088 | (1.070-1.103) |
| 0.020 | 1.086 | (1.069-1.101) |
| 0.050 | 1.086 | (1.069-1.102) |

c. Varying the length of economic crisis

We have simulated - with our models and our parameters – the effect of a different duration of the economic crisis in our estimates. Despite the fact that we used the most probable scenario of economic crisis^1^– due to the importance of the poverty rate variable in the regression models, we explored the effects of austerity measures with different forecast length of the period of poverty increase.

The Brazilian economy experienced one of its stronger economic crises in recent years with national GDP falling by more than 8% since mid-2014, but while economic recession in Brazil technically ended in late 2017, recovery is likely to be fragile given the depth of the economic contraction since 2014, the ongoing political crisis in the country, and continued increases in unemployment, income inequality and poverty are indicative of a persistent social and economic crisis.^14,15^.

Table E and Figure E show that, irrespective of the duration of poverty rates increases, the effect of austerity-induced ESF coverage on ACSC mortality will be very similar.

***Table E – ACSC mortality rate ratios between the ESF coverage scenarios and static ESF coverage for 2030 with varying intensities of economic crisis***

|  | **Shorter Economic crisis** | **Medium Economic crisis** | **Longer Economic crisis** |
| --- | --- | --- | --- |
|  |  |  |  |
| **Static ESF coverage (scenario 1)** | 1(Ref) | 1(Ref) | 1(Ref) |
| **Contracting ESF coverage (scenario 2)** | 1.055 | 1.057 | 1.061 |
|  | (1.040-1.071) | (1.042-1.073) | (1.046-1.077) |
| **Static ESF coverage (scenario 1)** | 1.083 | 1.086 | 1.089 |
|  | (1.067-1.093) | (1.069-1.102) | (1.072-1.103) |

*ESF - Estratégia Saúde da Família (Family Health Strategy); ACSC – ambulatory care sensitive conditions*

***Figure E – Three situations of economic crisis and varying poverty rates with ACSC mortality rates for the four ESF coverage scenarios***

**
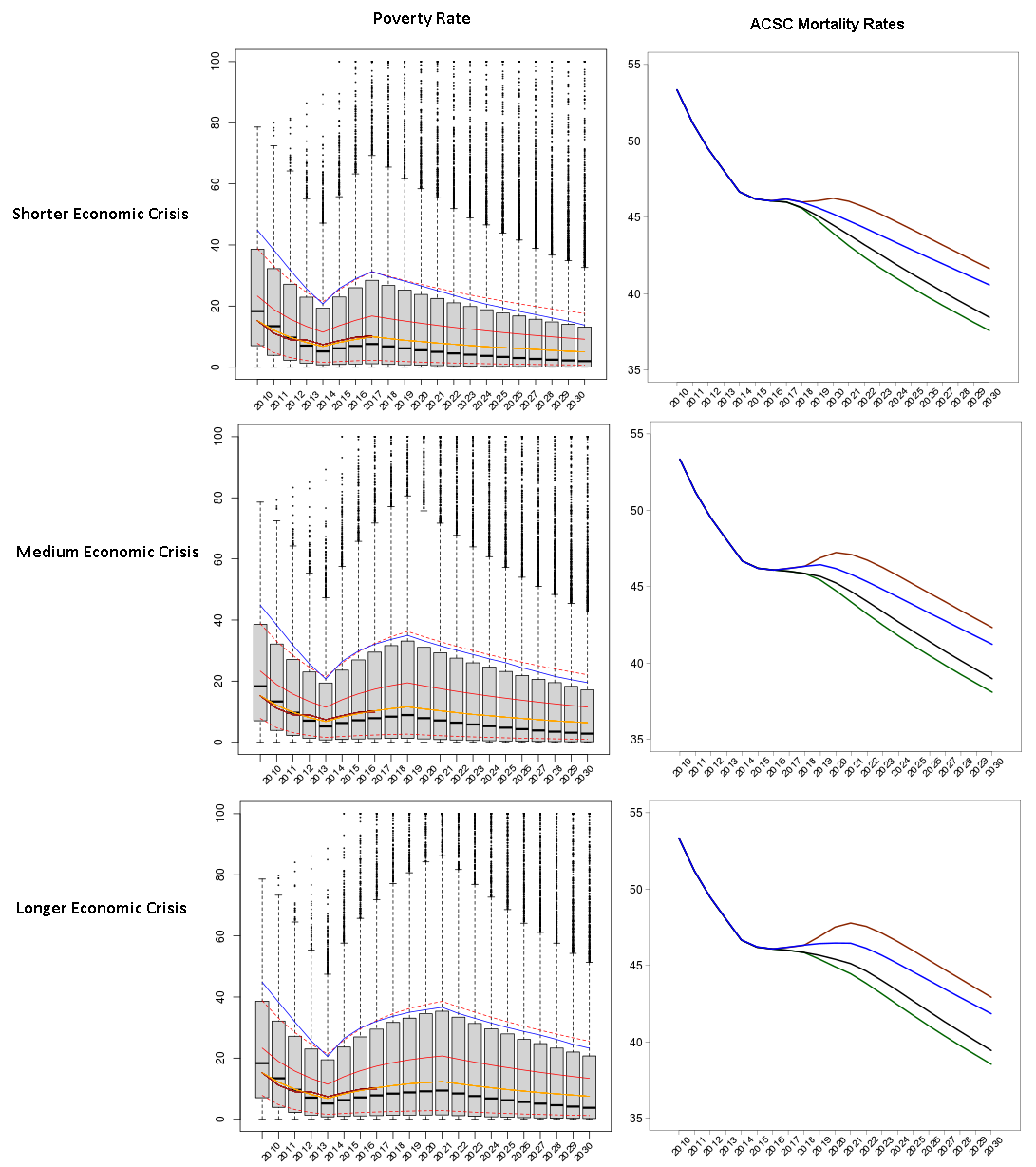
**

*ACSC mortality expressed per 100,000 inhabitants; the four ESF scenarios are (from highest to lowest ACSC mortality rates in 2030): ESF contraction and termination of Mais Médicos (scenario 3) (red line); ESF contraction (scenario 2) (blue line); static ESF coverage (scenario 1) (grey line); and UHC (scenario 4) (green line). ESF - Estratégia Saúde da Família (Family Health Strategy);*

d. Varying duration effects of the exposure variable

In addition to average municipal two year ESF coverage used in the retrospective evaluation^2^ and in the microsimulation models, a mean ESF average coverage of the last 4 years – used in previous ESF impact evaluations^4^, was also tested. Comparing the extremes of UHC (scenario 4) and ESF contraction and *Mais Médicos* termination (scenario 3), the difference in the ACSC mortality rate in 2030 would be 8.6% (95%CI:6.9%-10.2%), which is slightly lower than the 11.1% (95%CI: 9.5%-12.7%) reported in the main text when using two-year ESF coverage.

e. Stratifying the analysis according to poverty levels

A different effect size for ESF coverage was also tested according to the level of poverty of the municipality (dichotomized), showing no important difference with the results of the main analysis. Comparing the extremes of UHC (scenario 4) and ESF contraction and *Mais Médicos* termination (scenario 3), the rate ratio in ACSC mortality in 2030 would be 11.5% (95%CI:9.8%-13.1%) – highly comparable to the 11.1% (95%CI: 9.5%-12.7%) reported in the main text. Due to the incertitude in the heterogeneity of the poverty increase along the municipalities, the more conservative assumption of a similar increase (calibrated with World Bank estimates) has been chosen.

**Main limitations**

One limitation of the study is the uncertainty around the future macroeconomic scenarios in Brazil, due to the current extremely unstable political and economic situation, which creates uncertainty around the forecasting of poverty rates and the other independent variables. For that reason several scenarios have been simulated in sensitivity analyses which produced comparative findings.

Another limitation is that the modelling of austerity measures is focused on ESF as there is strong evidence that these policies confer protective effects for mortality rates.^2,3,6^ Our estimates of the impact of austerity measures on child health are probably conservative as they do not reflect constraints in other areas of public spending e.g. education, housing and other welfare programmes which have known impacts on poverty and health. Moreover, austerity measures recently enshrined in the constitution of Brazil means that public spending will only increase in line with inflation, which will not account for the demographic growth of the population, its ageing processes, and growing costs associated with new healthcare treatments and technologies.^11,12,13^.

**Sources of funding and their role**

The development of the model was funded by the Wellcome Trust Training Fellowships in Public Health and Tropical Medicine scheme (Grant reference number: 109949/Z/15/Z). CM is funded by a Research Professorship award from the National Institute for Health Research. The funding was not specific for the study described in this article. The funder had no role in study design, data collection, data analysis, data interpretation, writing of the report, or in the decision to submit this article for publication. All researchers´ decisions have been entirely independent from funders.

**2. SIMULATION OF FISCAL AUSTERITY**

Several simulations have been run on how fiscal austerity could affect the coverage of the Estratégia Saúde da Família (ESF).The most probable scenarios have been drawn from two technical notes from the governmental Institute of Applied Economic Research (IPEA), which estimated the impact of the Emenda Constitucional 95 (EC95) on the budget for Social Assistance policies ^16^ and for the National Health System ^17^ respectively.

EC95 limits yearly Federal Expenses for Social Assistance (SA) and Healthcare for the period 2017-2037 at the values of the previous year increased for inflation, so while it will slightly increase the budget each year, this increase would be insufficient to maintain present levels of services offered to the population.^13,14.15^ This will be due to 3 main reasons:

1. Population growth, which will be approximately 1% per year in the next years, reducing the amount of money per capita available both in SA and healthcare.

2. Population ageing, which in the Social Protection and in the Healthcare sector will increase budget needs - restricting resources for other programmes, including ESF.

3. Higher yearly costs for drugs and medical supplies and incorporation of new drugs and technologies, which will increase per capita expenses for healthcare.^18^

For the healthcare system (Figure F), or Unified Health System (Sistema Único de Saúde, SUS), the hypothetical effects of the EC95, if it was implemented in the period 2000-2015 - have been compared to the budget which has been applied during the same period and which allowed the maintenance and some expansions of healthcare services. It has to be considered that the SUS has suffered from chronic underfunding since its creation and austerity measures can only worsening this situation.^14,15,19,20,21^

The yearly ratios between EC95 and non-EC95 budgets have been calculated, and they have been fitted with an exponential decay formula obtaining an estimate of the percent change. This was estimated to be 5.5%. As shown above, several other percentage decreases were tested as sensitivity analysis.

***Figure F. Percent of the GDP for Healthcare According to Economic Austerity (EC95) or maintenance to pre-existing levels. The comparison is between the real spending in the period 2004-2015 and the simulated spending if EC95 was applied during the same period.***

**
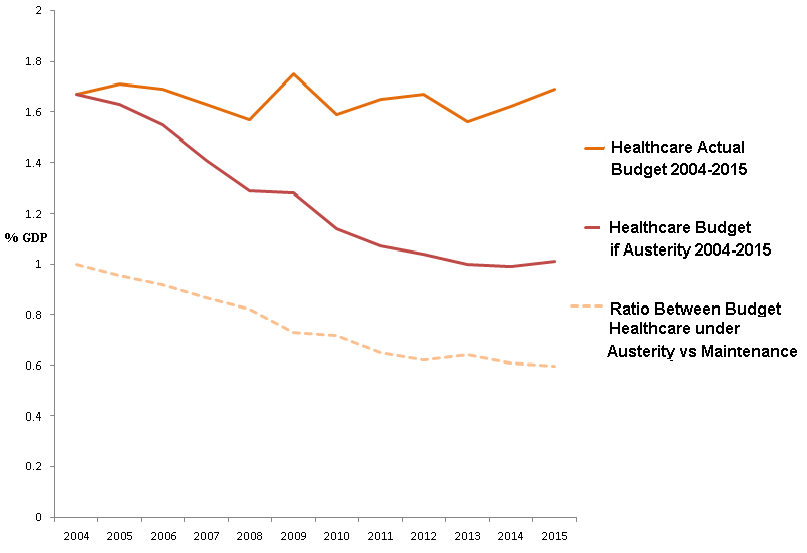
**

The methods for estimating the effect of austerity measures are similar to the one used in a recent paper on the effects of economic crisis and fiscal austerity on child mortality in Brazil, but these calculations have been focused on the healthcare budget instead of social assistance budget.^1^

Austerity measures are likely to affect a broad range of benefits and services offered under Social Assistance programmes and through the Health Care System. For this microsimulation exercise ESF impacts were modelled as there is robust evidence that these programmes confer protection against ACSC MR.

**3. AMBULATORY CARE SENSISTIVE CONDITIONS**

The mortality rate from ACSCs under-70 was the forecast variable. In the retrospective part analysis^2^ ACSC deaths were encoded based on a list published by the Brazilian Ministry of Health (and restricted to those aged
under 70 y) and ICD-10 codes reported on death certificates (Table F). ACSCs were
grouped by cause of death into infectious diseases, nutritional deficiencies and anaemia, and cardiovascular disease.

***Table F. Ambulatory-care-sensitive conditions with International Classification of Diseases (ICD-10) codes.***

| **Group** | **Condition** | **ICD-10 codes** |
| --- | --- | --- |
| **Infectious diseases** | **Vaccine-preventable diseases** |  |
|  | Tetanus | A33–A35 |
|  | Diphtheria | A36 |
|  | Whooping cough | A37 |
|  | Yellow fever | A95 |
|  | Acute hepatitis B | B16 |
|  | Measles | B05 |
|  | Rubella | B06 |
|  | Mumps | B26 |
|  | Haemophilus meningitis | G00.0 |
|  | Tuberculous meningitis | A17.0 |
|  | Miliary tuberculosis | A19 |
|  | **Preventable conditions** |  |
|  | Tuberculosis | A15–A16, A17.1–A17.9, A18 |
|  | Acute rheumatic fever | I00–I02 |
|  | Syphilis (early and late) | A51–A53 |
|  | Malaria | B50–B54 |
|  | Ascariasis | B77 |
|  | **Gastrointestinal infections and complications** |  |
|  | Intestinal infectious diseases | A00–A09 |
|  | Dehydration | E86 |
|  | **Infections of the ear, nose, and throat** |  |
|  | Otitis media | H66 |
|  | Acute upper respiratory infections | J00–J03, J06, J31 |
|  | **Bacterial pneumonias** | J13–J14, J15.3–J15.4, J15.8–J15.9, J18.1 |
|  | **Infections of the kidney and urinary tract** |  |
|  | Nephritis | N10–N12 |
|  | Cystitis | N30 |
|  | Urethritis and urethral syndrome | N34 |
|  | Urinary tract infection | N39.0 |
|  | **Diseases of the prenatal period and childbirth** |  |
|  | Urinary tract infection during pregnancy | O23 |
|  | Congenital syphilis | A50 |
|  | Congenital rubella | P35.0 |
|  | **Infections of skin & subcutaneous tissue** | A46, L01–L04, L08 |
|  | **Pelvic inflammatory disease** | N70–N73, N75–N76 |
| **Nutritional deficiencies** | **Anaemia** | D50 |
|  | **Nutritional deficiencies** |  |
|  | Malnutrition | E40–E46 |
|  | Other nutritional deficiencies | E50–E64 |
| **COPD and asthma** | **Asthma** | J45–J46 |
|  | **Diseases of the lower respiratory tract** |  |
|  | Bronchitis | J20, J21, J40–J42 |
|  | Emphysema | J43 |
|  | COPD | J44 |
|  | Bronchiectasis | J47 |
| **Cardiovascular disease** | **Hypertension** | I10–I11 |

| **Group** | **Condition** | **ICD-10 codes** |
| --- | --- | --- |
|  | **Angina** | I10 |
|  | **Heart failure** | I50, J81 |
|  | **Cerebrovascular disease** | I63–I67, I69, G45–G46 |
| **Diabetes** | **Diabetes mellitus** | E10–E14 |
| **Epilepsy** | **Epilepsy** | G40–G41 |
| **Gastric ulcers** | **Gastric ulcers** | K25–K28, K92.0, K92.1, K92.2 |

*Source: A list published by Alfradique et al.^22^ and developed with the Brazilian Ministry of Health.
COPD, chronic obstructive pulmonary disease.*

**REFERENCES**

1. Rasella D, Basu S, Hone T, Paes-Sousa R, Ocké-Reis CO, Millett C. Child morbidity and mortality associated with alternative policy responses to the economic crisis in Brazil: A nationwide microsimulation study. PLoS Med. 2018 May 22;15(5):e1002570

2.Hone T, Rasella D, Barreto ML, Majeed A, Millett C. Association between expansion of primary healthcare and racial inequalities in mortality amenable to primary care in Brazil: A national longitudinal analysis. PLoS Med. 2017;14(5): e1002306.

3. Rasella D, Harhay MO, Pamponet ML, Aquino R, Barreto ML. Impact of primary health care on mortality from heart and cerebrovascular diseases in Brazil: a nationwide analysis of longitudinal data. BMJ. 2014;349: g4014.

4. Rasella D, Aquino R, Santos CAT, Paes-Sousa R, Barreto ML. Effect of a conditional cash transfer programme on childhood mortality: a nationwide analysis of Brazilian municipalities. The Lancet. 2013;382(9886): 57-64.

5. Dourado I, Oliveira VB, Aquino R, Bonolo P, Lima-Costa MF, Medina MG, Mota E, Turci MA, Macinko J. Trends in primary health care-sensitive conditions in Brazil: the role of the Family Health Program (Project ICSAP-Brazil). Med Care. 2011 Jun;49(6):577-84

6. Hone T, Rasella D, Barreto M, Atun R, Majeed A, Millett C.Large Reductions In Amenable Mortality Associated With Brazil's Primary Care Expansion And Strong Health Governance. Health Aff (Millwood). 2017 Jan 1;36(1):149-158.

7. EPE, Ministerios de Minas e Energias. Cenários Macroeconômicos 2017-2030. Available at: http://www.epe.gov.br/sites-pt/publicacoes-dados-abertos/publicacoes/PublicacoesArquivos/publicacao-245/topico-261/DEA%20009-17%20-%20Cen%C3%A1rio%20macroecon%C3%B4mico_2017-2026_VF%5B1%5D.pdf

8. Ministério da Saúde Brasil. DATASUS. 2017. http://tabnet.datasus.gov.br/ (accessed 08/04 2017).

9. Fiebig D, Johar M, Forecasting with Micro Panels: The Case of Health Care Costs. Journal of Forecasting, 2016, 36:1-15

10. Baltagi H, Forecasting with panel data. Journal of Forecasting, 2008, 27:153-161.

11. Paiva AB, Mesquita ACS, Jaccoud L, Passos L. [The new tax regime and its implications for social assistance policy in Brazil.] [Portuguese]. Technical Note No.27. Brasilia, Brazil: Instituto de Pesquisa Econômica Aplicada (IPEA), 2016.

12. Vieira FS, Benevides RPdSe. [The impacts of the New Tax Regime for the financing of the Unified Health System and for the realization of the right to health in Brazill] [Portuguese]. Technical Note No.28. Brasilia, Brazil: Instituto de Pesquisa Econômica Aplicada (IPEA), 2016.

13. Rossi P, Dweck E. Impacts of the new fiscal regime on health and education. Cadernos de saude publica 2016; 32:12-15.

14. Massuda A, Hone T, Leles FAG, de Castro MC, Atun R. The Brazilian health system at crossroads: progress, crisis and resilience. BMJ Glob Health. 2018 Jul 3;3(4):e000829.

15. Doniec K, Dall'Alba R, King L. Brazil's health catastrophe in the making. Lancet. 2018 Jul 19.

16. Paiva AB, Mesquita ACS, Jaccoud L, Passos L. [The new tax regime and its implications for social assistance policy in Brazil.] [Portuguese]. Technical Note No.27. Brasilia, Brazil: Instituto de Pesquisa Econômica Aplicada (IPEA), 2016.

17. Vieira FS, Benevides RPdSe. [The impacts of the New Tax Regime for the financing of the Unified Health System and for the realization of the right to health in Brazill] [Portuguese]. [Portuguese] Technical Note No.28. Brasilia, Brazil: Instituto de Pesquisa Econômica Aplicada (IPEA), 2016.

18. Ocké-Reis CO, Marmor TR. The Brazilian national health system: an unfulfilled promise? Int J Health Plann Manage. 2010;25(4):318-29.

19.de Souza LEPF. The right to health in Brazil: A Constitutional guarantee threatened by fiscal austerity. J Public Health Policy. 2017 Nov;38(4):493-502.

20. Doniec K, Dall'Alba R, King L. Austerity threatens universal health coverage in Brazil. . Lancet. 2016 Aug 27;388(10047):867-8.

21. Marques R, Mendes A.[The financing of Basic Health Care and the Family Health Strategy in the Unified Health System]. [Portuguese] Saúde Debate, 2014, 38(103):900-916.

22. Alfradique ME, Bonolo PdF, Dourado I, Lima-Costa MF, Macinko J, Mendonca CS, et al. Ambulatory care sensitive hospitalizations: elaboration of Brazilian list as a tool for measuring health system performance (Project ICSAP–Brazil). Cad Saude Publica. 2009; 25(6):1337–49
